# Effects of Vitamin D Supplementation on CD4^+^ T Cell Subsets and mTOR Signaling Pathway in High-Fat-Diet-Induced Obese Mice

**DOI:** 10.3390/nu13030796

**Published:** 2021-02-28

**Authors:** Jeong Hee An, Da Hye Cho, Ga Young Lee, Min Su Kang, So Jeong Kim, Sung Nim Han

**Affiliations:** 1 Department of Food and Nutrition, College of Human Ecology, Seoul National University, Seoul 08826, Korea; don03174@snu.ac.kr (J.H.A.); likefnt@snu.ac.kr (D.H.C.); lgykiki90@snu.ac.kr (G.Y.L.); usnim17@snu.ac.kr (M.S.K.); gksdn3835@snu.ac.kr (S.J.K.); 2 Research Institute of Human Ecology, Seoul National University, Seoul 08826, Korea

**Keywords:** CD4^+^ T cell, vitamin D supplementation, mTOR pathway, obesity, HIF1α

## Abstract

Obesity is associated with an impaired balance of CD4^+^ T cell subsets. Both vitamin D and obesity have been reported to affect the mTOR pathway. In this study, we investigated the effects of vitamin D on CD4^+^ T cell subsets and the mTOR pathway. Ten-week-old male C57BL/6 mice were divided into four groups and fed diets with different fat (control or high-fat diets: CON or HFD) and vitamin D contents (vitamin D control or supplemented diets: vDC or vDS) for 12 weeks. T cells purified by negative selection were stimulated with anti-CD3/anti-CD28 mAbs and cultured for 48 h. The percentage of CD4^+^IL-17^+^ T cells was higher in the vDS than vDC groups. The CD4^+^CD25^+^Foxp3^+^ T cells percentage was higher in HFD than CON groups. The phospho-p70S6K/total-p70S6K ratio was lower in vDS than vDC, but the phospho-AKT/total-AKT ratio was higher in vDS than vDC groups. *Hif1α* mRNA levels were lower in vDS than vDC groups. These findings suggest HIF1α plays an important role in vitamin-D-mediated regulation of glucose metabolism in T cells, and dietary vitamin D supplementation may contribute to the maintenance of immune homeostasis by regulating the mTOR pathway in T cells.

## 1. Introduction

CD4^+^ T cells play an important role in orchestrating the adaptive immune responses to various pathogens [1]. Upon T-cell receptor (TCR) stimulation, naive CD4^+^ T cells are activated and differentiate into several CD4^+^ T subsets, which have specific effector functions depending on the cytokines present in the environment during their activation. CD4^+^ T cell subsets, including Th1, Th2, Th17, and Treg cells, can be distinguished by their cytokine production profiles and specific transcription factors [2].

The mechanistic target of rapamycin (mTOR) signaling pathway is a central regulator of cell metabolism, growth, proliferation, and survival [3]. Moreover, mTOR exists as two complexes, mTOR complex 1 (mTORC1) and mTOR complex 2 (mTORC2), which have distinct cellular actions and physiological functions. In T cells, mTOR can be activated by immune signals, nutrients, and environmental cues, and it regulates T-cell proliferation and CD4^+^ T-cell differentiation [4]. Although the effects of mTOR on CD4^+^ T cell differentiation have been investigated in many studies, the results are controversial. *Mtor*^-/-^ CD4^+^ T cells showed normal activation markers in response to TCR stimulation but failed to differentiate into Th1, Th2, and Th17 cells [5]. T cells depleted of RHEB, an upstream activator of mTORC1, failed to differentiate into Th1 and Th17 cells [5]. In contrast, deletion of RAPTOR, an essential subunit of mTORC1, resulted in impaired Th17 but normal Th1 differentiation [6]. Delgoffe et al. [7] reported that *Rictor*^-/-^ CD4^+^ T cells with selective inhibition of mTORC2 activity failed to differentiate into Th2 cells but successfully differentiated into Th1 and Th17 cells. However, Lee et al. [8] demonstrated that RICTOR-deficient T cells showed reduced Th1- and Th2-cell differentiation. Additionally, mTORC1 promoted glycolytic metabolism in effector CD4^+^ T cells through hypoxia-inducible factor 1-α (HIF1α) [9]. Pathogenic CD4^+^ T cells in autoimmune diseases are characterized by increased glucose uptake and the upregulation of glycolytic enzymes [10]. The inhibition of glucose metabolism in CD4^+^ T cells has been reported to improve clinical outcomes in inflammatory bowel disease and experimental autoimmune encephalomyelitis mice [11,12].

Obesity is known to be associated with immune dysfunction [13], characterized by increased T-cell numbers and a shift in CD4^+^ T cell subsets toward a pro-inflammatory phenotype [14]. In addition, a Th17/Treg imbalance could lead to increased insulin resistance [15]. The suggested mechanism for increased insulin resistance associated with an impaired Th17/Treg balance is an increase in inflammatory cytokine production, which induces the infiltration of M1 macrophages and glucose uptake inhibition [16,17,18]. In obese mice and humans, increased numbers of CD4^+^ T cells [19,20], Th1, and Th17 cells [21,22,23] and a decreased number of Treg cells [16,24] were observed in the peripheral blood and adipose tissue. However, an increased number and percentage of Treg cells have been shown to be associated with obesity in several studies [25,26]. Although the precise mechanistic explanations for the alteration in the number and population of CD4^+^ T cells seen with obesity remain unclear, mTOR activation by increased leptin may be involved [27].

Vitamin D can regulate the innate and adaptive immune systems, and many studies have demonstrated the effects of vitamin D on the differentiation and cellular metabolism of CD4^+^ T cells. Treatment with 1,25(OH)_2_D_3_ decreased the production of IFN-γ and IL-17 and differentiation into Th1 and Th17 cells in splenocyte and CD4^+^ T cells from mice [28,29], but increased IL-10 production and differentiation into Treg cells [30]. However, the effects of 1,25(OH)_2_D_3_ on the differentiation of Th2 cells are still controversial. Boonstra et al. [31] reported that 1,25(OH)_2_D_3_ treatment increased IL-4 production and *Gata3* mRNA levels in splenic T cells. Pichler et al. [32] demonstrated that 1,25(OH)_2_D_3_ treatment decreased the percentage of IL-4^+^CD4^+^ T cells. Furthermore, vitamin D has been reported to affect the mTOR pathway: in vitro treatment with vitamin D and vitamin D analogs decreased mTOR activity in osteoblast and human breast cancer cell line [33,34]. However, the precise mechanisms of mTOR pathway regulation by vitamin D remain unclear, and few studies have examined the effects of vitamin D supplementation on CD4^+^ T cell subsets or mTOR pathway in T cells in vivo.

Reportedly, there is a relationship between obesity and altered vitamin D metabolism. Low serum 25-hydroxy vitamin D levels have been observed in obese individuals [35,36], and a recent meta-analysis showed that vitamin D deficiency was significantly more prevalent in obese people [37]. Several mechanisms, such as the decreased synthesis of vitamin D in the skin due to reduced sun exposure and the sequestration of vitamin D in adipose tissue, have been proposed to lead to vitamin D deficiency in obese people [38,39]. Given the associations among vitamin D, obesity, and CD4^+^ T cells, it is worth examining whether the effects of vitamin D on CD4^+^ T cell subsets are influenced by obesity.

In this study, we investigated the effects of dietary vitamin D supplementation on CD4^+^ T cell subsets and the mTOR pathway in both lean control (CON) mice and high-fat diet (HFD)-induced obese mice. To achieve this purpose, we examined the expression of surface markers, cytokines, and genes involved in CD4^+^ T cell subsets, as well as the expression of proteins and genes related to the mTOR pathway and vitamin D metabolism in T cells.

## 2. Materials and Methods

### 2.1. Animals and Diets

Ten-week-old male C57BL/6N mice were purchased from Central Lab Animal Inc. (Seoul, Korea) and housed in a specific pathogen-free room at Seoul National University with an environmentally controlled temperature (23 ± 1 °C), relative humidity of 50 ± 10%, and a 12-h light/12-h dark cycle. After 7 days of acclimation, the mice were randomly assigned to four groups (*n* = 14–15 per each group) and fed experimental diets differing in fat and vitamin D content for 12 weeks. The diets were designed with 10% or 45% kcal fat (CON and HFD, respectively) and about 1000 or 10,000 IU vitamin D/kg diet (vDC and vDS, respectively) (CON-vDC, D12450H; CON-vDS, D17090501; HFD-vDC, D12451; HFD-vDS, D17090502; Research Diets, Inc., New Brunswick, NJ, USA). The compositions of the diets are shown in Table 1. The food and water were provided ad libitum. Food intake was measured four times per week, and body weight was recorded once a week. At the end of the experimental period, mice were fasted for 12 h and euthanized by CO_2_ asphyxiation. Blood was collected by cardiac puncture, and serum was separated by centrifugation at 3000 rpm for 20 min and stored at −80 °C. The spleen and white adipose tissue, including perirenal, intraperitoneal, epididymal, and subcutaneous fat, were collected. All procedures were approved by the Institutional Animal Care and Use Committee of Seoul National University (approval number: SNU-191007-3-3).

### 2.2. T Cell Isolation and Culture

The spleen was aseptically removed and placed into sterile RPMI 1640 (Lonza, Walkersville, MD, USA) medium supplemented with 100,000 U/L penicillin (GibcoBRL, Grand Island, NY, USA), 100 mg/L streptomycin (GibcoBRL), 25 mM HEPES (Sigma Aldrich, St. Louis, MO, USA), and 2 mM L-glutamine (GibcoBRL) (complete RPMI). A single-cell suspension of splenocytes was prepared by homogenizing the spleen, using sterile frosted glass slides (Thermo Fisher Scientific, Waltham, MA, USA). Tissue debris was removed via centrifugation, and red blood cells were lysed with Gey’s solution. T cells were purified by negative selection, using the Pan T cell isolation kit II (Miltenyi Biotec Inc., Auburn, CA, USA), according to the manufacturer’s instructions. The purified T cells were cultured in 24-well plates (2 × 10^6^ cells/well) with complete RPMI medium containing 10% fetal bovine serum (FBS) (GibcoBRL). T cells were then stimulated with plate-bound anti-CD3ε mAb (5 μg/mL, hamster anti-mouse, clone 145–2C11; BD Pharmingen, Franklin Lakes, NJ, USA) and soluble anti-CD28 mAb (2 μg/mL, hamster anti-mouse, clone 37.51; BD Pharmingen) and incubated for 48 h at 37 °C in humidified air with 5% CO_2_. After 48 h of incubation, the cells were collected for further analysis.

### 2.3. Serum 25(OH)D Concentration Measurement

Serum 25(OH)D concentration was measured by using a 25-Hydroxy Vitamin D^s^ EIA kit (Immunodiagnostic Systems Std, Boldon, UK), according to the manufacturer’s instructions.

### 2.4. Flow Cytometric Analysis

T cells were treated with GolgiPlug (2 μg/mL, BD Biosciences, Franklin Lakes, NJ, USA), 4 h prior to the end of the 48 h culture, to block the secretion of cytokines. Then, the T cells were collected and resuspended in fluorescence activating cell sorting (FACS) staining buffer (0.09% sodium azide, 1% FBS, 1 × PBS base) at 5 × 10^5^ cells/mL. A total of 5 × 10^5^ cells (per sample) were stained with APC-conjugated anti-mouse CD4 (BD Biosciences) and PE-conjugated anti-mouse CD25 (BD Biosciences) Abs for 30 min at 4 °C. For intracellular staining, T cells were fixed and permeabilized, using Foxp3/Transcription Factor Staining Buffer Set (Invitrogen, Carlsbad, CA, USA), and then stained with Alexa Fluor 488-conjugated anti-mouse Foxp3 (BD Biosciences) and PerCP-Cy^TM^ 5.5 anti-mouse IL-17A (BD Biosciences) Abs, for 30 min, at 4°C. After staining, the cells were washed and resuspended in a FACS-staining buffer with 4% formaldehyde, and then analyzed, using FACSLyric (BD Biosciences) and FlowJo software version 10 (BD Biosciences). Gating strategy of CD4^+^IL-17^+^ T cells and CD4^+^CD25^+^Foxp3^+^ T cells are provided in Supplementary Figure S1.

### 2.5. Quantification of Cytokine Production

After 48 h of incubation, the supernatant was collected and stored at −80 °C. The levels of IFN-γ and IL-4 produced by the T cells were determined by using mouse ELISA IFN-γ and IL-4 kits (BD Biosciences) according to the manufactures’ instructions. Absorbance was measured at 450 nm, with a microplate spectrophotometer (SpectraMax iD3, Molecular Devices, CA, USA).

### 2.6. Western Blot Analysis

Total protein was extracted from the T cells, using radioimmunoprecipitation assay buffer (50 mM Tris-HCl (pH 7.4), 1% NP-40, 0.25% sodium deoxycholate, 150 mM NaCl, 1 mM ethylenediaminetetraacetic acid, 1 mM phenylmethylsulfonyl fluoride, 1 mM sodium orthovanadate (Na3VO4), 1 mM sodium fluoride (NaF), 1 mM sodium pyrophosphate, 1 mM β-glycerophosphate, 10% glycerol, and a protease inhibitor cocktail tablet (Roche, Rotkreuz, Switzerland)). Protein lysates (25 µg) were electrophoresed on 13% sodium dodecyl sulfate-polyacrylamide gel electrophoresis, transferred to a polyvinylidene difluoride membrane, and blotted with phosphor-p70S6K (Thr389) (1:1000), total-p70S6K (1:2000), phospho-AKT (Ser473) (1:1000), total-AKT (1:2000), LC3I/II (1:1000), and P62 (1:1000) followed by horseradish peroxidase-conjugated anti-rabbit IgG (1:3000). All antibodies were purchased from Cell Signaling Technology (Danvers, MA, USA). Specific bands on the membrane were visualized with chemiluminescence luminol reagent (Santa Cruz Bio, Inc., Santa Cruz, CA, USA) and developed using a JP-33 automatic X-ray film processor (JPI Healthcare, Guro-gu, Seoul, Korea). The target bands were visualized and analyzed, using Quantity One 4.6 (Bio-Rad, Hercules, CA, USA).

### 2.7. RNA Extraction and Quantitative Real-Time PCR

Gene expression was measured in T cells at 48 h after TCR stimulation. Total RNA was extracted from the stimulated T cells, using RNAiso Plus (Takara Bio Inc., Otsu, Shiga, Japan), and cDNA was synthesized, using a PrimeScriptTM 1st strand cDNA synthesis kit (Takara Bio) with a thermal cycler (Applied Biosystems, Foster City, CA, USA). The quantitative real-time (q)PCR was conducted on a StepOne Real-time PCR system (Applied Biosystems). Each PCR reaction mixture contained synthesized cDNA, TB Green Premix Ex Taq, ROX reference dye (Takara Bio), and specific primers for the target gene. Table 2 lists the sequences of the primers used. The relative expression levels of the genes were calculated with the 2^-ΔΔCt^ method, using glyceraldehyde 3-phosphate dehydrogenase (*Gapdh*) as a housekeeping gene. 

### 2.8. Statistical Analysis

All statistical analyses were conducted by using SPSS Statistics version 25 (IBM SPSS Statistics, Chicago, IL, USA). Two-way analysis of variance (ANOVA) was used to evaluate the overall effects of the fat and vitamin D contents and the interaction, followed by Fisher’s least significant difference post hoc test, to compare differences among groups. If there was an interaction between variables, the data were analyzed with the Student’s *t*-test. All data are presented as means ± standard error of means (SEMs), and statistical significance was set at *p* < 0.05. 

## 3. Results

### 3.1. Body Weight, Weight Change, Adipose Tissue Weight, and Food Intake

There was no significant difference in body weight at week 0 among the groups. Body weight (*p* < 0.001), weight gain (*p* < 0.001), and white adipose tissue weight (*p* < 0.001) were higher in the HFD than CON groups at week 12 (Table 3). Vitamin D content had no significant impact on body weight or weight gain at week 12. The average energy intake was higher (*p* < 0.001) in the HFD groups compared with the CON groups. Vitamin D content in the HFD diet was 23% higher than the control diet, therefore, vitamin D intake was 21.6% higher in the HFD-vDS than the CON-vDS group (*p* < 0.001) and 19.9% higher in the HFD-vDC than the CON-vDC group (*p* < 0.001). 

### 3.2. Serum 25(OH)D Concentration 

**Figure 1:**
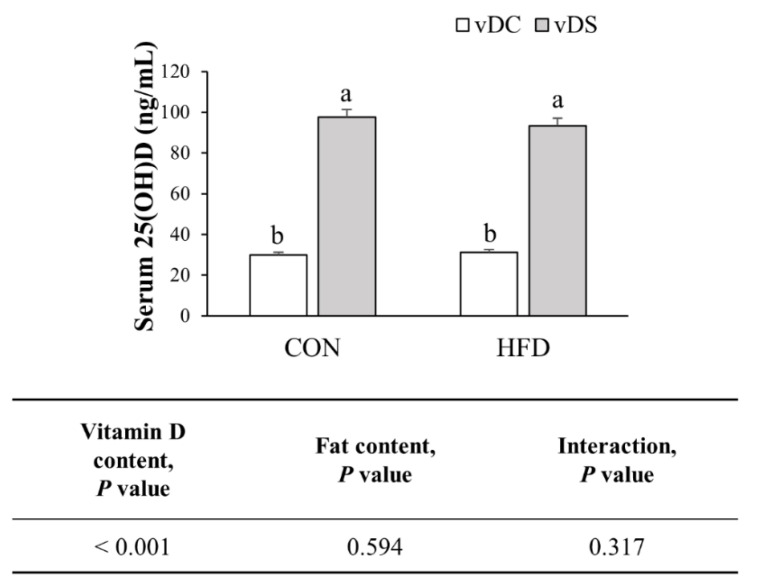
Serum 25(OH)D concentration. Data are presented as means ± SEMs, *n* = 9 per group. Two-way ANOVA was used to determine the significant effects of fat and vitamin D contents, and an interaction. ^a,b^ Different superscripts indicate significant difference (*p* < 0.05) among individual groups, by Fisher’s least significant difference multiple comparison test. CON-vDC, 10% kcal fat diet + vitamin D control; CON-vDS, 10% kcal fat diet + vitamin D supplemented; HFD-vDC, 45% kcal fat diet + vitamin D control; HFD-vDS, 45% kcal fat diet + vitamin D supplemented.

Serum 25(OH)D levels were higher in the vitamin-D-supplemented groups (*p* < 0.001) (Figure 1). Serum 25(OH)D levels were 227.8% higher in the CON-vDS than the CON-vDC group (*p* < 0.001) and 199.7% higher in the HFD-vDS than the HFD-vDC group (*p* < 0.001). However, the fat content had no significant effect on the serum 25(OH)D levels despite the higher average vitamin D intake in the HFD groups, as compared with the CON groups. 

### 3.3. Population of CD4^+^IL-17^+^ T cells and CD4^+^CD25^+^Foxp3^+^ T Cells

**Figure 2:**
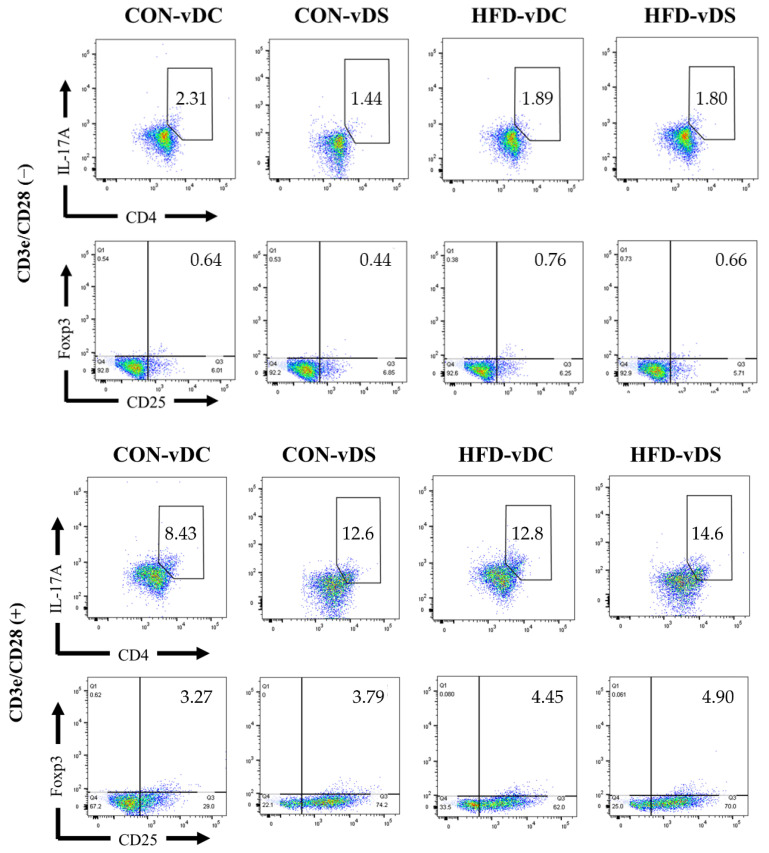
Populations of CD4^+^IL-17^+^ T cells and CD4^+^CD25^+^Foxp3^+^ T cells (dot plots). The populations of CD4^+^IL-17^+^ T cells and CD4^+^CD25^+^Foxp3^+^ T cells are presented as dot plots of the FlowJo analyses. CON-vDC, 10% kcal fat diet + vitamin D control; CON-vDS, 10% kcal fat diet + vitamin D supplemented; HFD-vDC, 45% kcal fat diet + vitamin D control; HFD-vDS, 45% kcal fat diet + vitamin D supplemented.

To investigate the effects of vitamin D and fat contents on the populations of CD4^+^ T cell subsets, we analyzed the percentages of CD4^+^IL-17^+^ T cells and CD4^+^CD25^+^Foxp3^+^ T cells, and CD4^+^IL-17^+^ T cells/CD4^+^CD25^+^Foxp3^+^ T cells ratio (Table 4). In unstimulated T cells, the percentage of CD4^+^IL-17^+^ T cells was unaffected by vitamin D and fat contents, but the percentage of CD4^+^CD25^+^Foxp3^+^ T cells was affected by the vitamin D content (*p* < 0.05) of the diets. There were 61.4% more CD4^+^CD25^+^Foxp3^+^ T cells in the HFD-vDS group, compared with the HFD-vDC group (*p* < 0.05), whereas there was no difference between the CON-vDS and CON-vDC groups. In stimulated T cells, the vitamin D content affected the percentage of CD4^+^IL-17^+^ T cells (*p* < 0.05). The percentage of CD4^+^IL-17^+^ T cells in the CON-vDS group tended to be higher than that in the CON-vDC group (36.7% higher, *p* = 0.08), but there was no difference between the HFD-vDS and HFD-vDC groups. Overall, the percentage of CD4^+^IL-17^+^ T cells tended to be higher in the HFD groups compared with the CON groups (*p* = 0.084), and there was a higher percentage of CD4^+^CD25^+^Foxp3^+^ T cells in the HFD than CON groups (*p* < 0.05). However, CD4^+^IL-17^+^ T cells/CD4^+^CD25^+^Foxp3^+^ T cells ratio was unaffected by the vitamin D or fat contents. Representative populations of CD4^+^IL-17^+^ T cells and CD4^+^CD25^+^Foxp3^+^ T cells are shown as dot plots in Figure 2.

### 3.4. Production of IFN-γ and IL-4 by T-Cells

**Figure 3:**
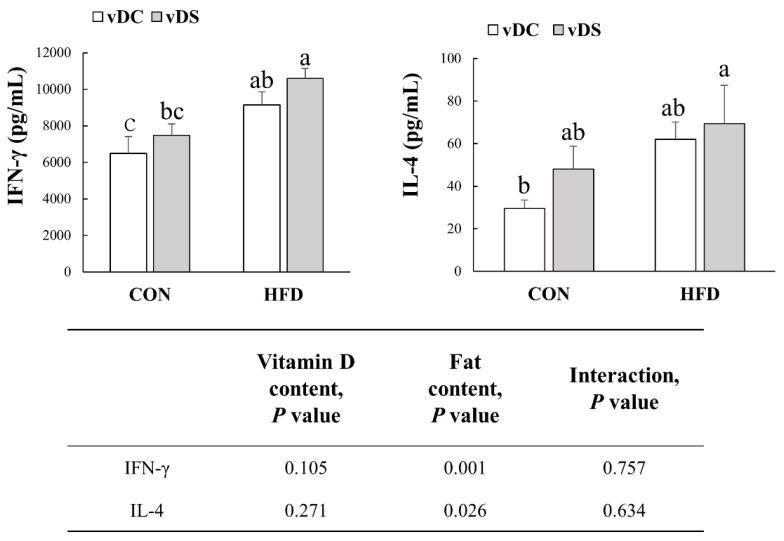
Production of IFN-γ and IL-4 by T cells. Splenic T cells were stimulated with anti-CD3/anti-CD28 mAbs for 48 h and supernatants were harvested to determine the amount of IFN-γ and IL-4 by ELISA. Data are presented as means ± SEMs, *n* = 7 per group. Two-way ANOVA was used to determine the significant effects of fat and vitamin D contents, and an interaction. ^a,b^ Different superscripts indicate significant difference (*p* < 0.05) among individual groups, by Fisher’s least significant difference multiple comparison test. CON-vDC, 10% kcal fat diet + vitamin D control; CON-vDS, 10% kcal fat diet + vitamin D supplemented; HFD-vDC, 45% kcal fat diet + vitamin D control; HFD-vDS, 45% kcal fat diet + vitamin D supplemented.

IFN-γ production by T cells was higher in the HFD groups than the CON groups (*p* < 0.001) (Figure 3). IFN-γ levels were 41.2% higher in the HFD-vDC group compared with the CON-vDC group (*p* < 0.05) and 39.9% higher in the HFD-vDS than CON-vDS group (*p* < 0.01). Overall, IL-4 levels were higher in the HFD groups (*p* < 0.05). Vitamin D content did not have a significant effect on either the IFN-γ or IL-4 levels produced by T cells.

### 3.5. Expression of Proteins Related to mTOR Pathway in T Cells

**Figure 4:**
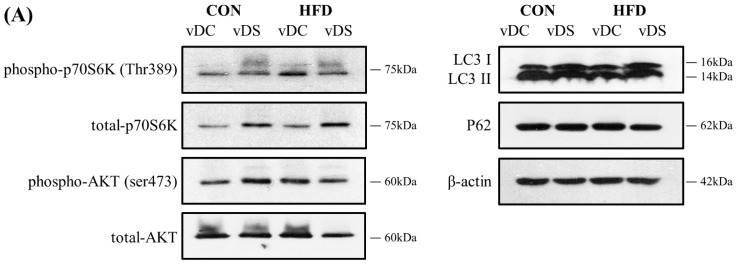

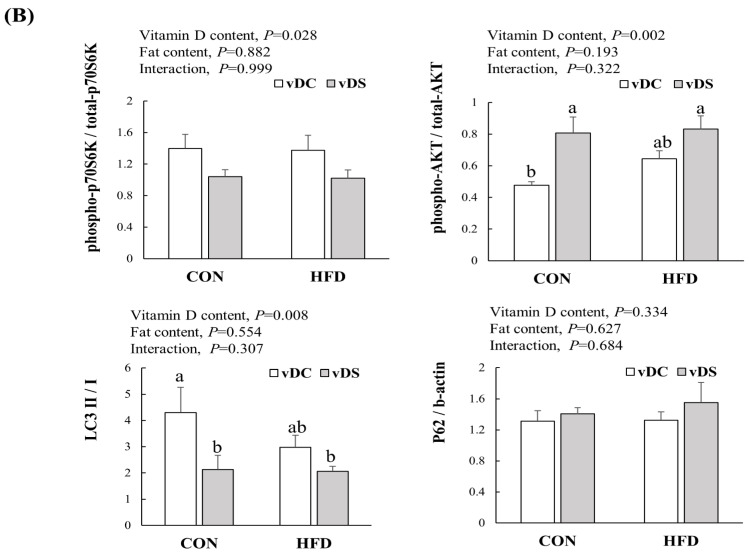
Levels of proteins related with mTOR pathway in T cells. Splenic T cells were stimulated with anti-CD3/anti-CD28 mAbs for 48 h, and the expression levels of p70S6K, AKT, LC3, and P62 were determined by Western blot. (**A**) Representative Immunoblots of mTOR pathway proteins. (**B**) Data are presented as means ± SEMs, *n* = 5 per group. Two-way ANOVA was used to determine the significant effects of fat and vitamin D contents, and an interaction. ^a,b^ Different superscripts indicate significant difference (*p* < 0.05) among individual groups, by Fisher’s least significant difference multiple comparison test. CON-vDC, 10% kcal fat diet + vitamin D control; CON-vDS, 10% kcal fat diet + vitamin D supplemented; HFD-vDC, 45% kcal fat diet + vitamin D control; HFD-vDS, 45% kcal fat diet + vitamin D supplemented.

The expression levels of phospho-p70S6K, total-p70S6K, phospho-AKT, total-AKT, LC3 II/I, and P62 were determined to investigate the effects of vitamin D and fat contents on the mTOR pathway in T cells (Figure 4). The amount of fat did not significantly affect the expression levels of these proteins, whereas the vitamin D content affected the expression levels of p70S6K, AKT, and LC3 II/I. Overall, the ratio of phospho-p70S6K/total-p70S6K, which is an indicator of mTORC1 activity, was lower in the vDS groups than the vDC groups (*p* < 0.05). The ratio of phospho-AKT/total-AKT, an indicator of mTORC2 activity, was higher in the vDS groups than the vDC groups (*p* < 0.01). The ratio of phospho-AKT/total-AKT was 68.8% higher in the CON-vDS group compared with the CON-vDC group (*p* < 0.05), whereas there was no difference between the HFD-vDS and HFD-vDC groups. 

Overall, the ratio of LC3 II/I was lower in the vDS than vDC groups (*p* < 0.01). However, the fat content did not have a significant effect on the ratio of LC3 II/I. The ratio of LC3 II/I was 101.9% higher in the CON-vDC group compared with the CON-vDS group (*p* < 0.05), but there was no significant difference between the HFD-vDS and HFD-vDC groups. P62 protein expression levels were not significantly affected by the vitamin D or fat content of the diets.

### 3.6. The mRNA Levels of Genes Involved in Vitamin D Metabolism in T Cells

**Figure 5:**
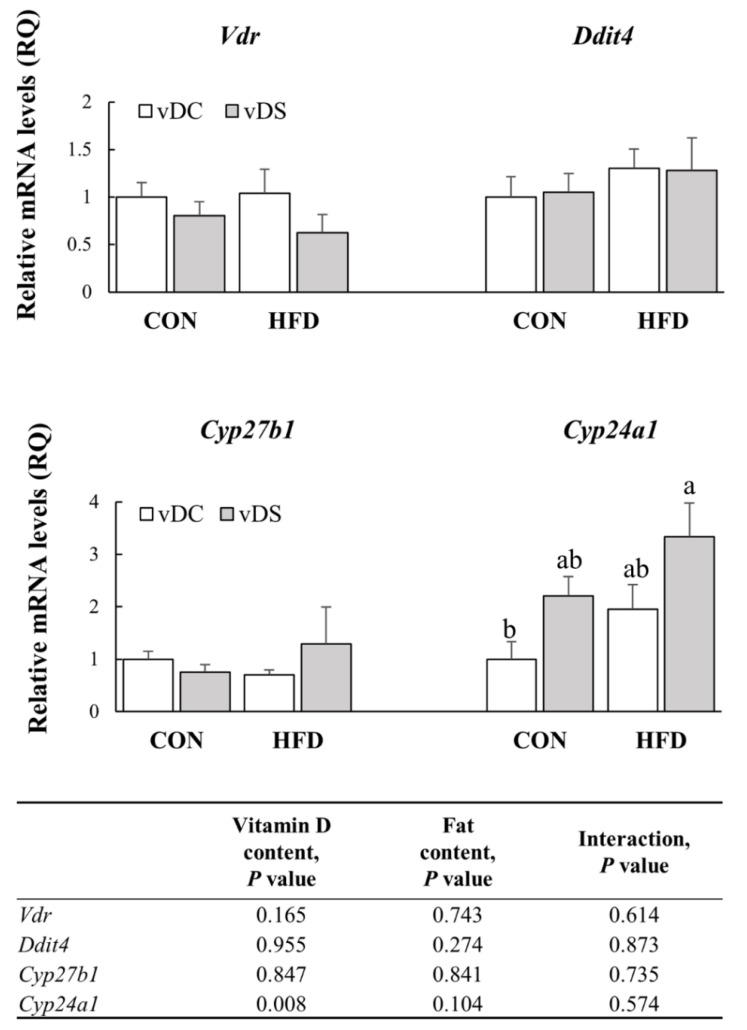
mRNA levels of *Vdr*, *Ddit4*, *Cyp27b1,* and *Cyp24a1* in T cells. Splenic T cells were stimulated with anti-CD3/anti-CD28 mAbs for 48 h, and the mRNA levels of *Vdr*, *Ddit4*, *Cyp27b1* and *Cyp24a1* were determined by qPCR. Data are presented as means ± SEMs, *n* = 7–9 per group. Two-way ANOVA was used to determine the significant effects of fat and vitamin D contents, and an interaction. ^a,b^ Different superscripts indicate significant difference (*p* < 0.05) among individual groups by Fisher’s least significant difference multiple comparison test. All values were normalized to the levels of housekeeping gene *Gapdh* and expressed as relative mRNA levels, compared with the average levels of the CON-vDC group. CON-vDC, 10% kcal fat diet + vitamin D control; CON-vDS, 10% kcal fat diet + vitamin D supplemented; HFD-vDC, 45% kcal fat diet + vitamin D control; HFD-vDS, 45% kcal fat diet + vitamin D supplemented.

To investigate the effects of dietary vitamin D and fat contents on vitamin D metabolism in T cells, the mRNA levels of *Vdr, Ddit4, Cyp27b1*, and *Cyp24a1* were determined (Figure 5). Neither the vitamin D nor fat content affected *Vdr, Ddit4*, and *Cyp27b1* expression in T cells. The *Cyp24a1* mRNA levels were higher in the vDS groups than the vDC groups (*p* < 0.01); however, they were not affected by dietary fat.

### 3.7. The mRNA Levels of Genes Involved in Th1 and Th2 Differentiation

**Figure 6:**
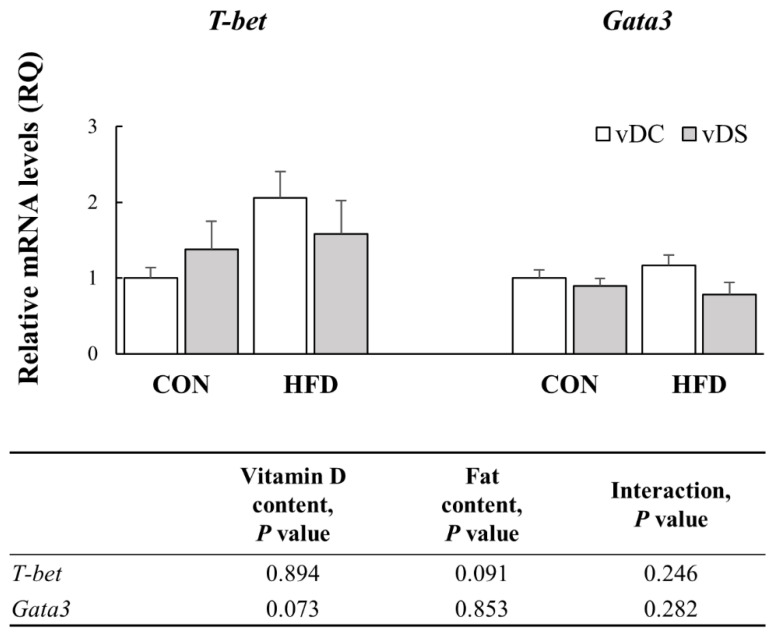
mRNA levels of *T-bet* and *Gata3* in T cells. Splenic T cells were stimulated with anti-CD3/anti-CD28 mAbs for 48 h, and the mRNA levels of *T-bet* and *Gata3* were determined by qPCR. Data are presented as means ± SEMs, *n* = 7–9 per group. Two-way ANOVA was used to determine the significant effects of fat and vitamin D contents, and an interaction. All values were normalized to the levels of housekeeping gene *Gapdh* and expressed as relative mRNA levels compared with the average levels of the CON-vDC group. CON-vDC, 10% kcal fat diet + vitamin D control; CON-vDS, 10% kcal fat diet + vitamin D supplemented; HFD-vDC, 45% kcal fat diet + vitamin D control; HFD-vDS, 45% kcal fat diet + vitamin D supplemented.

*T-bet* and *Gata3* mRNA levels were determined to investigate the effects of vitamin D and fat contents on Th1 and Th2 cell differentiation (Figure 6). The amount of fat tended to affect *T-bet* expression (*p* = 0.091). *T-bet* mRNA levels were 105.6% higher in the HFD-vDC group, compared with the CON-vDC group (*p* < 0.05), whereas there was no difference between the CON-vDS and HFD-vDS groups. Overall, *Gata3* mRNA levels tended to be lower in the vDS groups, compared with the vDC groups (*p* = 0.073), but the fat content did not significantly affect *Gata3* mRNA levels.

### 3.8. The mRNA Levels of Genes Involved in Glycolytic Pathway

**Figure 7:**
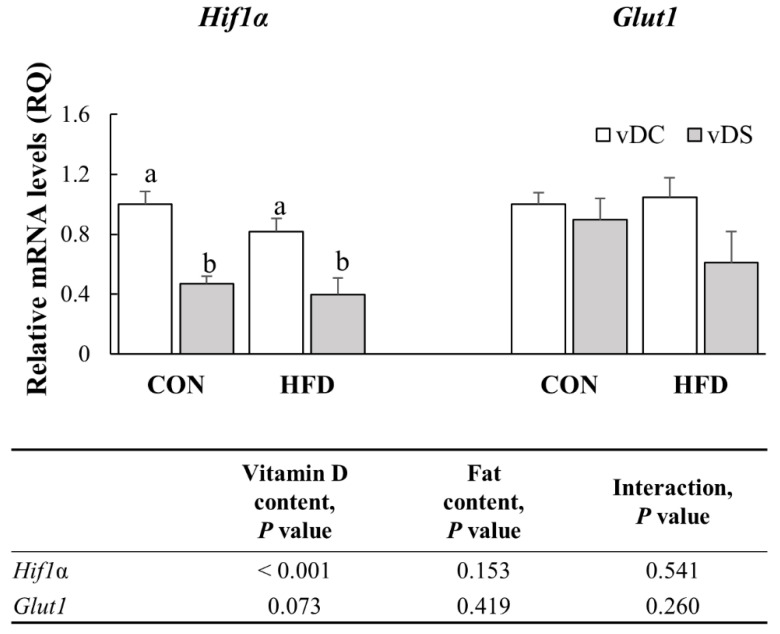
mRNA levels of *Hif1α* and *Glut1* in T cells. Splenic T cells were stimulated with anti-CD3/anti-CD28 mAbs for 48 h, and the mRNA levels of *Hif1α* and *Glut1* were determined by qPCR. Data are presented as means ± SEMs, *n* = 7–9 per group. Two-way ANOVA was used to determine the significant effects of fat and vitamin D contents, and an interaction. ^a,b^ Different superscripts indicate significant difference (*p* < 0.05) among individual groups, by Fisher’s least significant difference multiple comparison test. All values were normalized to the levels of housekeeping gene *Gapdh* and expressed as relative mRNA levels, compared with the average levels of the CON-vDC group. CON-vDC, 10% kcal fat diet + vitamin D control; CON-vDS, 10% kcal fat diet + vitamin D supplemented; HFD-vDC, 45% kcal fat diet + vitamin D control; HFD-vDS, 45% kcal fat diet + vitamin D supplemented.

To examine the effects of vitamin D and fat contents on the glycolytic pathway of T cells, we measured *Hif1α* and *Glut1* mRNA levels (Figure 7). *Hif1α* mRNA levels were higher in the vDC groups compared with the vDS groups (*p* < 0.001), and the levels were 113.0% higher in the CON-vDC than the CON-vDS group (*p* < 0.001) and 107.0% higher in the HFD-vDC than HFD-vDS (*p* < 0.01). Overall, *Glut1* mRNA levels tended to be lower in the vDS groups compared with the vDC groups (*p* = 0.073). However, the amount of fat in the diet did not significantly affect *Hif1α* and *Glut1* mRNA levels. 

## 4. Discussion

This study showed that vitamin D supplementation had differential effects on mTORC1 and mTORC2 activity in the T cells of both lean and obese mice. The ratio of phospho-p70S6K/total-p70S6K was significantly lower and the ratio of phospho-AKT/total-AKT was significantly higher in T cells from vitamin-D-supplemented mice. Moreover, *Hif1α* mRNA levels were lower, and *Glut1* mRNA levels tended to be lower in T cells from vitamin-D-supplemented mice. Given that mTORC1 promotes glucose metabolism through HIF1α, the lower expression of *Hif1α* seemed to be due to the lower mTORC1 activity, which was downregulated by vitamin D supplementation. 

Several cross-sectional studies have consistently reported the association between obesity and lower serum 25(OH)D levels [40,41]. A recent meta-analysis reported a significantly higher prevalence of vitamin D deficiency in obese people [37]. Despite the 21.6% higher average vitamin D intake (IU/day) in the HFD-vDS group compared with the CON-vDS group in this study, there was no difference in serum 25(OH)D levels, and this may be partially explained by the dysregulation of vitamin D metabolism in obesity. Park et al. [42] reported that high-fat-diet-induced obesity resulted in large amounts of vitamin D being stored in the liver and adipose tissue, which might contribute to lower serum 25(OH)D levels when vitamin D intake was at supplementary levels. 

We demonstrated that mTORC1 and mTORC2 activities in T cells were differentially regulated by vitamin D supplementation. Only a few studies have investigated the effects of vitamin D on mTOR activity. DDIT4 was shown to inhibit mTOR function by mediating tuberous sclerosis complex 1/2, an upstream regulator of mTOR [43]. Lisse et al. [33] reported that 1,25(OH)_2_D_3_ increased the expression levels of DDIT4 and suppressed the expression of phospho-pS6K1 in osteoblasts. According to O’Kelly et al. [34], in vitro treatment with vitamin D analogs decreased the expression of phospho-AKT and phospho-p70S6K in a human breast cancer cell line. They concluded that vitamin D directly inhibits both mTORC1 and mTORC2 activities. In this study, the ratio of phospho-p70S6K/total-p70S6K was significantly lower, whereas the ratio of phospho-AKT/total-AKT was significantly higher, in the vDS than vDC groups. These results indicate that vitamin D supplementation downregulates mTORC1 activity and upregulates mTORC2 activity in T cells. However, *Ddit4* mRNA levels were unaffected by vitamin D supplementation. Recently, it was reported that microRNA expression levels and DNA methylation markers related to the mTOR pathway are modulated by vitamin D in rat T cells [44]. Vitamin D was postulated to downregulate the mTOR pathway in T cells through an epigenetic mechanism. Further research is necessary to investigate the precise mechanisms by which vitamin D regulates the mTOR signaling pathway in T cells. 

Rapamycin directly inhibits mTORC1 activity through the formation of complexes with the 12-kDa FK506-binding protein (FKBP12). In addition, rapamycin can also inhibit mTORC2 activity in a manner dependent on the dose and length of exposure [45]. Rapamycin and rapamycin analogs have been widely used as immunosuppressants for preventing transplant rejection, but the non-selective inhibition of mTOR activity reportedly impairs glucose homeostasis and increases insulin resistance [46,47,48,49]. Lamming et al. [46] explained that impaired glucose homeostasis was due to the chronic inhibition of mTORC2 caused by long-term rapamycin use and high-dose rapamycin treatment. Thus, to reduce the side effects associated with suppressed mTORC2 activity, several studies were conducted to identify selective mTORC1 inhibitors. Schreiber et al. [50] conducted a fasting glucose tolerance test after two weeks of intraperitoneal injection of DL001, a selective mTORC1 inhibitor. The area under the curve (AUC) for the rapamycin-treated mice was 33% higher than that of the control mice, whereas the AUC of the DL001-treated mice was the same as that of the control mice and glucose homeostasis was unimpaired. In the present study, we demonstrated that vitamin D supplementation selectively inhibited mTORC1 activity in mouse splenic T cells. The results from this study suggest that vitamin D supplementation has mTOR inhibitory effects without impairing glucose homeostasis.

An appropriate Th17/Treg balance is important for the suppression of excessive immune responses and the maintenance of immune homeostasis [22,51]. Obesity has been shown to affect the differentiation of Th17 and Treg cells; however, the results differed depending on the tissues or experimental methods used. The percentage of CD4^+^IL-17^+^ T cells in adipose tissue increased with obesity [22,52], and the percentage of CD4^+^IL-17^+^ cells in the spleen was higher in obese mice (60% kcal fat, 10 weeks) compared with control mice [53]. The percentage of CD4^+^Foxp3^+^ T cells in the intestinal mucosa was lower in obese (60% fat, 12 weeks) than control mice [54]. However, other studies have shown serum IL-10 levels and CD4^+^Foxp3^+^ T cells in adipose tissue to be higher in obese than lean subjects [25,26]. In this study, the percentage of CD4^+^IL-17^+^ T cells tended to be higher and that of CD4^+^CD25^+^Foxp3^+^ T cells was higher in purified T cells from the HFD groups compared with the CON groups; however, the CD4^+^IL-17^+^ T cells/CD4^+^CD25^+^Foxp3^+^ T cells ratio was unaffected by obesity. In a glucose tolerance test, IL-17-deficient obese mice showed enhanced glucose tolerance compared with wild-type obese mice [17]. In a previous study, the number of Treg cells negatively correlated with the number of pro-inflammatory M1-macrophages in adipose tissue [16], and Chen et al. [18] suggested that Treg cells inhibit M1-macrophage infiltration into the tissue. Metformin is widely used to improve glucose tolerance in type 2 diabetes mellitus patients. Metformin was shown to shift the impaired Th17/Treg balance in obese mice toward Treg cells, and these anti-inflammatory effects were associated with inhibited mTORC1 activity [55,56,57]. We observed that dietary vitamin D supplementation downregulated mTORC1 activity in T cells. Lower 25(OH)D levels have been seen in type 2 diabetes mellitus patients [58], and there appears to be a strong inverse association between low 25(OH)D levels and diabetes prevalence [59]. Although we did not observe the effects of obesity on the Th17/Treg balance in this study, vitamin D supplementation might contribute to improving the insulin resistance related to the obesity-induced Th17/Treg imbalance.

The percentage of CD4^+^IL-17^+^ T cells was higher in the vitamin-D-supplemented groups in this study. However, vitamin D did not significantly affect the percentage of CD4^+^CD25^+^Foxp3^+^ T cells or the CD4^+^IL-17^+^ T cells/CD4^+^CD25^+^Foxp3^+^ T cells ratio. Treatment with 10 nM 1,25(OH)_2_D_3_ decreased the percentage of IL-17^+^ T cells in the mouse spleen and human peripheral blood mononuclear cells (PBMC) [29,60]; whereas treatment with 10 nM 1,25(OH)_2_D_3_ increased IL-10-secreting CD4^+^ T cells by inducing tolerogenic dendritic cells [61] and upregulated *Foxp3* mRNA expression in PBMC [62]. According to Fleet et al. [63], the serum 1,25(OH)_2_D levels in mice fed 10,000 IU vitamin D/kg diet for 10 weeks were 0.2 to 0.25 nM. In a study conducted by our laboratory (not published), serum 1,25(OH)_2_D levels in mice fed 10,000 IU vitamin D/kg diet for 12 weeks were 0.4 to 0.5 nM, which are much lower than the levels in the studies in which the in vitro effects of 1,25(OH)_2_D_3_ were observed. In addition, the differentiation of CD4^+^ T cells is affected by the surrounding environment in which the CD4^+^ T cells are activated. Therefore, the effects of dietary vitamin D supplementation may not have been observed ex vivo because the T cells were cultured in 10% FBS/cRPMI without vitamin D treatment for 48 h. Previous studies by others used additional cytokine treatment to induce differentiation. In this study, CD4^+^ T cell subsets in stimulated T cells were confirmed, using anti-CD3/anti-CD28 mAbs; thus, different results from previous might be related to stimulation methods.

In this study, *Hif1α* mRNA levels were lower and *Glut1* mRNA levels tended to be lower in the vitamin-D-supplemented groups than the control groups. HIF1α is downstream of mTORC1 and is a critical regulator of glucose metabolism [9]. Shi et al. [11] reported that rapamycin treatment suppressed *Hif1α*, *Glut1*, and *Mct4* expression, and T cells from Hif1α^-/-^ mice expressed significantly lower mRNA levels of glycolytic enzymes. In addition, T cells depleted of RHEB and RAPTOR showed diminished glycolytic activity [64,65]. The regulation of metabolic pathways in immune cells plays an important role in immune functions [66]. In particular, pathogenic CD4^+^ T cells in autoimmune disease are characterized by increased glucose uptake and the upregulation of glycolytic enzymes. Thus, the regulation of glucose metabolism is considered a potential therapeutic target for T cell-mediated diseases [10]. Macintyre et al. [12] reported that, when colitis was induced, mice with a Glut1^-/-^ T cells did not undergo significant weight loss and showed less severe colitis compared with wild-type mice. In vitro treatment with 2-deoxyglucose decreased histological scores and alleviated disease severity in the EAE mice [11]. In this study, dietary vitamin D supplementation downregulated HIF1α, suggesting that vitamin D may affect glycolysis. Therefore, our results potentially provide a partial explanation for the underlying mechanisms controlling the effectiveness of vitamin D supplementation in T cell-mediated diseases.

The active form of vitamin D, 1,25(OH)_2_D, regulates various immune responses through binding with VDR. However, serum 1,25(OH)_2_D levels are tightly regulated within narrow limits under normal condition [67]. It has been reported that the 25(OH)D-1α-hydroxylase CYP27B1, which converts 25(OH)D to 1,25(OH)_2_D, is expressed on immune cells, including T cells [68]. Moreover, amount of 1,25(OH)_2_D locally synthesized was regulated by serum 25(OH)D levels [69]. Kongsbak et al. [70] confirmed that activated CD4^+^ T cells had the ability to convert 25(OH)D to 1,25(OH)_2_D and produced significantly higher amount of 1,25(OH)_2_D, compared with unstimulated CD4^+^ T cells. Thus, it could be inferred that high serum 25(OH)D levels due to vitamin D supplementation in this study might have affected the function and metabolism of CD4^+^ T cells by increasing local 1,25(OH)_2_D_3_ production. 

## 5. Conclusions

In conclusion, dietary vitamin D supplementation differentially regulated mTORC1 and mTORC2 activities in T cells in both control mice and HFD-induced obese mice. Vitamin D supplementation downregulated mTORC1 activity and upregulated mTORC2 activity, as well as lowering *Hif1α* mRNA levels. Since HIF1α functions as a critical regulator of glucose metabolism, HIF1α may play an important role in the vitamin-D-mediated regulation of glucose metabolism in T cells, and dietary vitamin D supplementation may contribute to the maintenance of immune homeostasis by regulating the mTOR pathway in T cells.

## Figures and Tables

**Table 1:** Composition of the experimental diets

	CON (10% kcal fat)		HFD (45% kcal Fat)	
	vDC	vDS	vDC	vDS
	(948 IU/kg Diet)	(9477 IU/kg Diet)	(1165 IU/kg Diet)	(11652 IU/kg Diet)
Casein, 30 Mesh (g)	200	200	200	200
L-Cystine (g)	3	3	3	3
Corn Starch (g)	452.2	452.2	72.8	72.8
Maltodextrin 10 (g)	75	75	100	100
Sucrose (g)	172.8	172.8	172.8	172.8
Cellulose, BW200 (g)	50	50	50	50
Soybean Oil (g)	25	25	25	25
Lard (g)	20	20	177.5	177.5
Mineral Mix (g) ^1^	10	10	10	10
DiCalcium Phosphate (g)	13	13	13	13
Calcium Carbonate (g)	5.5	5.5	5.5	5.5
Potassium Citrate (g)	16.5	16.5	16.5	16.5
Vitamin Mix (g) ^2^	10	10	10	10
Vitamin D3 (g),	0	0.09	0	0.09
(100,000 IU/g)				
Choline Bitartrate (g)	2	2	2	2
FD&C Yellow Dye No. 5 (g)	0.04	0	0	0.025
FD&C Red Dye No. 40 (g)	0.01	0	0.05	0
FD&C Blue Dye No. 1 (g)	0	0.5	0	0.025
Total (g)	1055.05	1055.14	858.15	858.24
kcal/g diet	3.85	3.84	4.73	4.73

Resource: Research Diets, Inc., New Brunswick, NJ, USA; ^1^ 10 g of Mineral Mix (Research Diets, Inc., #S10026) provides 1.0 g of Na, 1.6 g of Cl, 1.6 g of Mg, 0.33 g of S, 59 g of Mn, 37 mg of Fe, 29 mg of Zn, 6.0 mg of Cu, 2.0 mg of Cr, 1.6 mg of Mo, 0.16 mg of Se, 0.9 mg of Fl, 0.2 mg of I, and 3.99 g of sucrose; ^2^ 10 g of Vitamin Mix (Research Diets, Inc., #V10001) provides 4000 IU of vitamin A, 1000 IU of vitamin D3, 50 IU of vitamin E, 0.5 mg of menadione, 0.2 mg of biotin, 10 µg of vitamin B12, 2 mg of folic acid, 30 mg of niacin, 16 mg of pantothenic acid, 7 mg of vitamin B6, 6 mg of vitamin B2, 6 mg of vitamin B1, and 9.78 g of sucrose. CON, control; HFD, high-fat diet; vDC, vitamin D control 1000 IU vitamin D/kg diet; vDS, 10,000 IU vitamin D/kg diet.

**Table 2:** Sequences of primers in for quantitative real-time PCR.

Genes	Forward Primer	Reverse Primer
*Vdr*	5’-GGGATGATGGGTAGGTTGTG-3’	5’-GGAAGAGGGTAGAGGGCAGA-3’
*Ddit4*	5’-TGTAACCAGGGACCAAGGAA-3’	5’-GTGTGTGGAGCAAGGCAAG-3’
*Cyp27b1*	5’-GACGATGTTGGCTGTCTTCC-3’	5’-ATCTCTTCCCTTCGGCTTTG-3’
*Cyp24a1*	5’-TCCCTGAGTAATGGGCTTTG-3’	5’-CACGGTAGGCTGCTGAGATT-3’
*T-bet*	5’-AATCGACAACAACCCCTTTG-3’	5’-AACTGTGTTCCCGAGGTGTC-3’
*Gata3*	5’-GAACCGCCCCTTATCAAG-3’	5’-CAGGATGTCCCTGCTCTCCTT-3’
*Hif1α*	5’-CTTGAAAAAGGGAGCCATCA-3’	5’-ACAGCCTCACCAGACAGAGC-3’
*Glut1*	5’-CTGGACCTCAAACTTCATTGTGGG-3’	5’-GGGTGTCTTGTCACTTTGGCTGG-3’
*Gapdh*	5’-GGAGAAACCTGCCAAGTA-3’	5’-AAGAGTGGGAGTTGCTGTTG-3’

*Vdr*, vitamin D receptor; *Ddit4*, DNA-damage-inducible transcript 4; *Cyp27b1*, Cytochrome P450 family 27 subfamily B member 1; *Cyp24a1*, Cytochrome P450 family 24 subfamily A member 1; *T-bet*, T-box transcription factor 21; *Gata3*, GATA binding protein 3; *Hif1α*, Hypoxia-inducible factor 1-alpha; *Glut1*, Glucose transporter 1; *Gapdh*, Glyceraldehyde-3-phosphate dehydrogenase.

**Table 3:** Body weight, weight change, adipose tissue weight, and food intake.

	CON		HFD		Vitamin D Content, *p*-Value	Fat Content, *p*-Value	Interaction, *p*-Value
	vDC	vDS	vDC	vDS			
Body weight at week 0 (g)	23.4 ± 0.4	23.7 ± 0.3	23.7 ± 0.3	23.9 ± 0.2	0.342	0.481	0.910
Body weight at week 12 (g)	31.5 ± 0.6 ^b^	31.4 ± 0.7 ^b^	41.3 ± 0.8 ^a^	42.5 ± 0.8 ^a^	0.428	<0.001	0.344
Weight gain (g)	8.1 ± 0.6 ^b^	7.7 ± 0.8 ^b^	17.6 ± 0.8 ^a^	18.7 ± 0.8 ^a^	0.616	<0.001	0.340
WAT weight (g)	3.5 ± 0.4 ^b^	3.1 ± 0.3 ^b^	7.4 ± 0.3 ^a^	7.5 ± 0.3 ^a^	0.567	<0.001	0.433
Average food intake (g/day)	2.6 ± 0.03 ^a,b^	2.6 ± 0.03 ^a^	2.5 ± 0.03 ^b^	2.6 ± 0.03 ^a^	0.001	0.078	0.491
Average energy Intake (kcal/day)	9.8 ± 0.1 ^c^	10.1 ± 0.1 ^c^	11.7 ± 0.1 ^b^	12.3 ± 0.1 ^a^	0.001	<0.001	0.275
Average vitamin D intake (IU/day)	2.4 ± 0.03 ^d^	24.9 ± 0.3 ^b^	2.9 ± 0.03 ^c^	30.3 ± 0.3 ^a^	<0.001	<0.001	<0.001

Data are presented as means ± SEMs, *n* = 14–15 per group. Two-way ANOVA was used to determine the significant effects of fat and vitamin D contents, and an interaction. ^a,b,c^ Different superscripts indicate significant difference (*p* < 0.05) among individual groups, by Fisher’s least significant difference multiple comparison test. CON-vDC, 10% kcal fat diet + vitamin D control; CON-vDS, 10% kcal fat diet + vitamin D supplemented; HFD-vDC, 45% kcal fat diet + vitamin D control; HFD-vDS, 45% kcal fat diet + vitamin D supplemented. WAT weight includes perirenal, intraperitoneal, epididymal, and subcutaneous fat.

**Table 4:** Populations of CD4^+^IL-17^+^ T cells and CD4^+^CD25^+^Foxp3^+^ T cells.

		CON		HFD		Vitamin D Content, *p*-Value	Fat Content, *p*-Value	Interaction, *p*-Value
		vDC	vDS	vDC	vDS			
CD3e CD28 (−)	CD4^+^IL-17^+^ T cells (%)	2.16 ± 0.37	1.89 ± 0.18	1.69 ± 0.36	2.00 ± 0.29	0.952	0.575	0.370
	CD4^+^CD25^+^Foxp3^+^ T cells (%)	0.66 ± 0.05 ^a,b^	0.74 ± 0.11 ^a^	0.44 ± 0.07 ^b^	0.71 ± 0.08 ^a^	0.037	0.118	0.226
	CD4^+^IL-17^+^ T cells / CD4^+^CD25^+^Foxp3^+^ T cells (ratio)	3.25 ± 0.50	2.96 ± 0.64	4.65 ± 1.43	3.29 ± 0.74	0.380	0.360	0.568
CD3e CD28 (+)	CD4^+^IL-17^+^ T cells (%)	9.83 ± 0.97 ^b^	13.44 ± 1.68 ^a^	12.80 ± 0.71 ^a,b^	14.53 ± 1.09 ^a^	0.026	0.084	0.412
	CD4^+^CD25^+^Foxp3^+^ T cells (%)	3.65 ± 0.46 ^b^	4.30 ± 0.43 ^a,b^	4.68 ± 0.30 ^a,b^	5.06 ± 0.35 ^a^	0.164	0.022	0.550
	CD4^+^IL-17^+^ T cells / CD4^+^CD25^+^Foxp3^+^ T cells (ratio)	2.78 ± 0.15	3.28 ± 0.51	2.87 ± 0.35	2.96 ± 0.30	0.383	0.739	0.555

Data are presented as means ± SEMs, *n* = 6–7 per group. Two-way ANOVA was used to determine the significant effects of fat and vitamin D contents, and an interaction. ^a,b^ Different superscripts indicate significant difference (*p* < 0.05) among individual groups, by Fisher’s least significant difference multiple comparison test. CON-vDC, 10% kcal fat diet + vitamin D control; CON-vDS, 10% kcal fat diet + vitamin D supplemented; HFD-vDC, 45% kcal fat diet + vitamin D control; HFD-vDS, 45% kcal fat diet + vitamin D supplemented.

## Data Availability

The data presented in this study are available on request from the corresponding author.

## Supplementary Materials

The following is available online, at https://www.mdpi.com/2072-6643/13/3/796/s1. Figure S1: Gating strategy for analysis of CD4^+^IL-17^+^ T cells and CD4^+^CD25^+^Foxp3^+^ T cells.
