# Supplementary material for: Effects of Vitamin D Supplementation on CD4+ T Cell Subsets and mTOR Signaling Pathway in High-Fat-Diet-Induced Obese Mice
Source: Nutrients. 2021 Feb 28;13(3):796. doi: 10.3390/nu13030796 (PMC7997284; doi:10.3390/nu13030796)
Supplement: Supplementary file 1 [file nutrients-13-00796-s001.pdf]

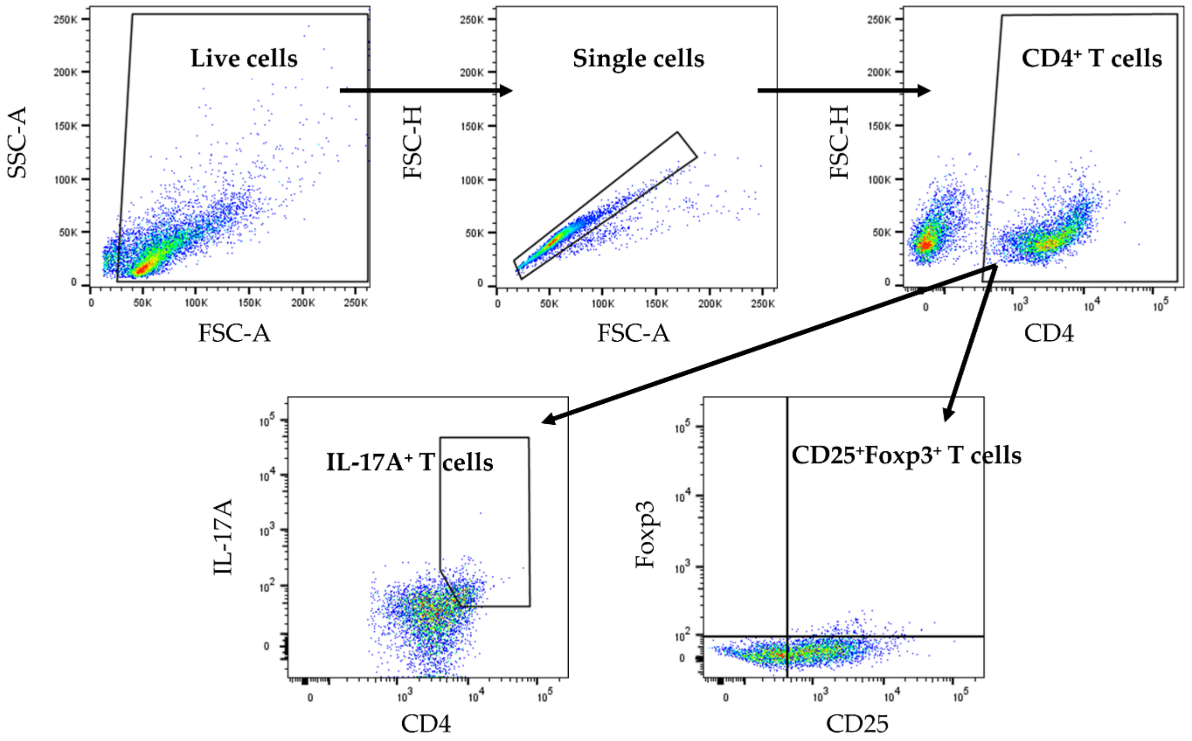

Figure S1. Gating strategy for analysis of CD4$^+$IL-17$^+$ T cells and CD4$^+$CD25$^+$Foxp3$^+$ T cells.

Cell were gated based on size using FSC-A and SSC-A to eliminate dead cells and debris. Single cells were gated using FSC-A and FSC-H. CD4$^+$ T cells were selected for further analysis. Subsequently, CD4$^+$ T cell was followed by examination of expression of IL-17 PerCP-Cy™ 5.5 and double expression of CD25 PE and Foxp3 Alexa Flour 488.
